# Capacity building and mentorship among pan-Canadian early career researchers in community-based primary health care

**DOI:** 10.1017/S1463423619000938

**Published:** 2020-02-06

**Authors:** Kathryn Nicholson, Rebecca Ganann, Sue Bookey-Bassett, Lisa Garland Baird, Anna Garnett, Zack Marshall, Anum Irfan Khan, Melissa Pirrie, Maxime Sasseville, Ali Ben Charif, Marie-Ève Poitras, Grace Kyoon-Achan, Émilie Dionne, Kasra Hassani, Moira Stewart

**Affiliations:** 1 Department of Epidemiology & Biostatistics, Western University, London, Ontario, Canada; 2 School of Nursing, McMaster University, Hamilton, Ontario, Canada; 3 Daphne Cockwell School of Nursing, Ryerson University, Toronto, Ontario, Canada; 4 Faculty of Nursing, University of Prince Edward Island, Charlottetown, Prince Edward Island, Canada; 5 Arthur Labatt Family School of Nursing, Western University, London, Ontario, Canada; 6 School of Social Work, McGill University, Montréal, Québec, Canada; 7 Institute of Health Policy, Management and Evaluation, University of Toronto, Toronto, Ontario, Canada; 8 Department of Family Medicine, McMaster University, Hamilton, Ontario, Canada; 9 Department of Health Sciences, Université du Québec à Chicoutimi, Chicoutimi, Québec, Canada; 10 Centre de recherche sur les soins et les services de première ligne (CERSSPL), Université Laval, Québec City, Québec, Canada; 11 Health and Social Services Systems, Knowledge Translation and Implementation Component of the Québec SPOR-SUPPORT Unit, Université Laval, Québec, Canada; 12 Département de médecine de famille et de médecine d’urgence, Faculté de médecine et des sciences de la santé, Université de Sherbrooke, Saguenay, Québec, Canada; 13 Rady Faculty of Health Sciences, University of Manitoba, Winnipeg, Manitoba, Canada; 14 Saint-Mary’s Research Centre & Department of Family Medicine, McGill University, Montreal, Québec, Canada; 15 School of Nursing and Centre for Health Services and Policy Research, University of British Columbia, Vancouver, British Columbia, Canada; 16 Centre for Studies in Family Medicine, Western University, London, Ontario, Canada

**Keywords:** capacity building, community-based primary health care, early career researchers, graduate students, interdisciplinary, mentorship, research trainees

## Abstract

**Aim:**

To describe activities and outcomes of a cross-team capacity building strategy that took place over a five-year funding period within the broader context of 12 community-based primary health care (CBPHC) teams.

**Background:**

In 2013, the Canadian Institutes of Health Research funded 12 CBPHC Teams (12-Teams) to conduct innovative cross-jurisdictional research to improve the delivery of high-quality CBPHC to Canadians. This signature initiative also aimed to enhance CBPHC research capacity among an interdisciplinary group of trainees, facilitated by a collaboration between a capacity building committee led by senior researchers and a trainee-led working group.

**Methods:**

After the committee and working group were established, capacity building activities were organized based on needs and interests identified by trainees of the CBPHC Teams. This paper presents a summary of the activities accomplished, as well as the outcomes reported through an online semistructured survey completed by the trainees toward the end of the five-year funding period. This survey was designed to capture the capacity building and mentorship activities that trainees either had experienced or would like to experience in the future. Descriptive and thematic analyses were conducted based on survey responses, and these findings were compared with the existing core competencies in the literature.

**Findings:**

Since 2013, nine webinars and three online workshops were hosted by trainees and senior researchers, respectively. Many of the CBPHC Teams provided exposure for trainees to innovative methods, CBPHC content, and showcased trainee research. A total of 27 trainees from 10 of the 12-Teams responded to the survey (41.5%). Trainees identified key areas of benefit from their involvement in this initiative: skills training, networking opportunities, and academic productivity. Trainees identified gaps in research and professional skill development, indicating areas for further improvement in capacity building programs, particularly for trainees to play a more active role in their education and preparation.

## Background

Primary health care systems around the world are going through reforms to adapt to the changing needs of the populations and their workforce [Macinko *et al.*, 2007; Canadian Institutes of Health Research (CIHR), 2013]. The term ‘community-based primary health care’ (CBPHC) emphasizes this important evolution to a more inclusive person-centered approach to primary health care delivered by multidisciplinary providers in diverse community-based settings. Research and innovation play a critical role in helping primary health care adapt to the increased need for complex management for chronic conditions and toward the vision of interdisciplinary team-based primary care homes. Effective training and mentorship for the next generation of CBPHC researchers are key for this movement to continue, and it is important for interdisciplinary and transdisciplinary research capacity (Choi and Pak, 2006) to be developed strategically in the field of CBPHC.

Research mentoring programs and experiences have become increasingly recognized as important by those engaged in health research capacity building (Bennett *et al.*, 2010; Stewart *et al.*, 2010; 2014). As a result, many studies have explored the role of mentorship in research training to learn about strategies for successful mentoring relationships, to understand the characteristics and actions of effective mentors and mentees, and to identify the characteristics of successful and failed mentoring relationships (Sambunjak *et al.*, 2006; Detsky and Baerlocher, 2007; Straus *et al.*, 2014). The findings demonstrate that successful mentoring relationships use a reciprocal model based on mutual respect and shared common values, with clear communication of needs, goals and expectations, and the planning of adequate time and methods for meaningful connection between mentor and mentee (Lach *et al.*, 2013). Whereas unsuccessful relationships are often characterized by poor communication, a lack of commitment to the mentorship, personality differences, conflicts of interest, and/or a mentor’s lack of experience (Straus *et al.*, 2014). Incorporating these findings in the development and implementation of research training programs and initiatives to build CBPHC researchers’ capacity building programs is influential to their success.

Over the last 15 years, several important funding initiatives have been implemented to address the need to build primary health care researcher capacity. For example, the Transdisciplinary Understanding and Training on Research–Primary Health Care (TUTOR-PHC) program has been funded since 2003 to address this recognized need (Stewart *et al.*, 2010). This transdisciplinary research capacity building program trains health care researchers and decision-makers from diverse disciplines and many geographic locations across Canada and abroad (Stewart *et al.*, 2010) to develop interdisciplinary approaches to CBPHC research that actively integrate a variety of approaches to ensure positive outcomes (Choi and Pak, 2006). Other organizations with a focus on health services and policy research have developed core competencies that have helped frame educational programs to prepare the next generation of leaders. Specifically, leaders in health services research have recommended a diverse array of competencies ranging from study design and data analysis to project management and knowledge translation (Bornstein *et al.*, 2018; Burgess *et al.*, 2018). Not as clear in such research mentorship programs are the roles to be played by the trainees themselves. While trainees receive guidance and teaching from their mentors, effective and meaningful capacity for responding adequately to the dynamic nature of CBPHC requires that trainees play a more active role in their education and preparation (Zea and Belgrave, 2009). For example, peer-to-peer mentorship and advances in information technology provide suitable platforms for geographically dispersed trainees to be more connected and to form a supportive community.

In Spring 2013, the CIHR and its provincial partners funded a five-year Community-Based Primary Health Care Signature Initiative to support highly innovative approaches to improving the delivery of appropriate and high-quality CBPHC to Canadians (CIHR, 2013; Ben Charif *et al.*, 2018; Wong *et al.*, 2018; Kendall *et al.*, 2018). The CBPHC Signature Initiative supported 12 cross-jurisdictional and multidisciplinary research teams (12-Teams) with programs of research across Canada, which aimed to achieve four main objectives: (1) develop and compare innovative models for CBPHC delivery within Canada or internationally; (2) catalyze effective knowledge translation approaches to improve delivery of CBPHC in Canada or internationally; (3) evaluate and improve the impact of innovations for CBPHC; and (4) build interdisciplinary and interprofessional capacity for the generation, synthesis, and application of CBPHC research (CIHR, 2013). The 12-Teams were mandated to cross-collaborate and produce knowledge beyond what would be feasible by any one team, including collection of data and reporting on a set of agreed common indicators (Wong *et al.*, 2018).

**Figure 1.**
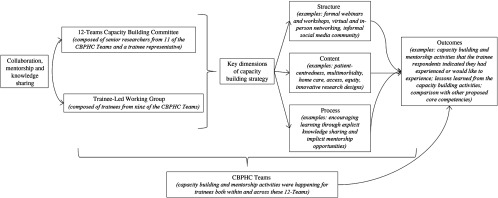
The structure and relationship of the capacity building strategy by the 12-Teams capacity building committee and the trainee-led working group, within the broader context of the CBPHC Teams

The 12-Teams included a large and diverse group of ‘trainees’, defined as individuals being trained at the undergraduate, graduate, postdoctoral, or professional level or individuals who were early career researchers. These individuals could also be completing their academic training, while assisting a CBPHC Team as a research coordinator, research assistant, or research associate. In line with their cross-team activities, the 12-Teams’ principal investigators initiated a capacity building committee and a trainee-led working group. The role of the capacity building committee was to provide mentorship for the working group as they organized activities, and this work took place within the broader context of the teams to which the trainees belonged (Figure 1). This paper specifically reports on the activities and outcomes of the capacity building strategy for CBPHC trainees.

## Methods

### Formation of the committee and the working group

Senior researchers from 11 of the 12 teams, as well as a 12-Teams trainee representative, formed the 12-Teams capacity building committee, beginning their activity immediately after the funding period began in 2013. One of the 12-Teams did not participate in trainee capacity building but instead focused on building capacity in the community in which their research was based. A principal investigator of 1 of the 12-Teams (M.S.) led the committee and requested participation of at least one senior researcher from the remaining teams interested in capacity building initiatives throughout the duration of the CBPHC funding period. The mandate of this committee was to organize educational events and activities for trainees, as well as to host trainee networking events at relevant academic conferences.

In early 2015, the 12-Teams capacity building committee recognized the need for a trainee-led working group for two main reasons: to assist with the development and implementation of events that were more relevant to the needs and interests of trainees and to provide opportunities for capacity building and leadership development among trainees during this process. The trainee representative who was a member of the capacity building committee (K.N.) assumed the leadership role in this new trainee-led working group. The capacity building committee then circulated an invitation email to a total of 85 CBPHC active trainees for them to join the working group. Twelve trainees from seven of the CBPHC Teams responded and formed the working group. These trainees represented diversity in disciplines, training levels, and geographic locations. With this membership, the working group functioned in parallel to the capacity building committee in planning, implementing, and evaluating its capacity building activities. The mandate of the working group was to facilitate educational activities for trainees: to encourage networking between trainees and other members of the 12-Teams; to share information relevant to trainees (e.g., CBPHC-related conferences, awards and career opportunities); and to increase trainee presence and involvement at conferences related to primary health care (e.g., North American Primary Care Research Group Annual Meeting, Canadian Association of Health Services and Policy Research Conference, and TUTOR-PHC Alumni Symposium). An online needs assessment survey of the trainees conducted in Summer 2015 guided the mandate of both the capacity building committee and the trainee-led working group to respond to the needs and interests of the CBPHC trainees (data available upon request). This needs assessment survey was used to determine interest in delivering an online webinar by the respondents about their research topics and preferred topics and formats of capacity building sessions. Respondents indicated an interest in the opportunity to share their research and a preference for online and recorded workshops about career building and advanced research methodologies.

## Data collection

In Summer 2017, the committee administered an online semistructured survey to identify activities and outcomes of capacity building and mentorship efforts as part of the CBPHC Signature Initiative. The survey was developed by the trainee-led working group and included both open- and closed-ended questions. The survey was posted using Google Forms, and responses were anonymized to protect participant identity. The invitation included all active CBPHC trainees. The 12-Teams trainee membership (and therefore the sampling frame) changed over time, from 85 trainees in Summer 2015 to 65 trainees in Summer 2017 due to completion of education programs or completion of affiliation with CBPHC Team. The sampling frame included a range of disciplines, training levels (undergraduate, graduate, postdoctoral, and early career), geographic locations, and all CBPHC Teams. The working group members pretested the survey, and the final version was administered via personalized emails and reminder emails to increase response rates (Dillman *et al.*, 2014). To further encourage trainee participation, senior researchers or the principal investigators from the CBPHC Teams sent emails to their respective trainees. The online survey remained opened for a two-month period to allow sufficient time for completion by interested trainee respondents.

## Data analysis

K.N. and Z.M. conducted the quantitative descriptive analyses for the closed-ended survey questions to explore trends pertaining to the characteristics of respondents and response distribution across questions. The thematic analysis of open-ended survey questions (conducted by A.I.K. and Z. M.) explored respondent perspectives on the perceived benefits of their involvement, unaddressed needs, and topics for future training sessions (Braun and Clarke, 2006; Green and Thorogood, 2009; Vaismoradi *et al.*, 2013). Coding of the data conducted by A.I.K. identified common ideas emerging from participant responses that were then categorized into themes as trends emerged (Braun and Clarke, 2006). A.I.K. and K.N. reviewed the themes to ensure appropriate categorization, mutual exclusion, and exhaustivity. All authors validated an overview of themes and key ideas under each emerging theme (Braun and Clarke, 2006). Due to the limited number of responses, no statistical software was needed for these analyses. Instead, the core competencies recently identified by the CIHR Canadian Health Services and Policy Research Alliance (Bornstein *et al.*, 2018; Burgess *et al.*, 2018) were used as a framework to contextualize the results from the thematic analysis. This was done by comparing the core competencies identified by the CIHR Canadian Health Services and Research Policy Alliance with the trainee responses.

## Findings

### Characteristics and capacity building activities

The characteristics of the trainee-led working group are presented in Table 1, and the details of the completed capacity building activities are presented in Table 2. Since 2015 when the working group was established, the membership of the working group included a total of seven CBPHC Teams and several disciplines, jurisdictions, and institutions. As seen in Figure 1, the capacity building activities were purposefully planned using three complementary dimensions: structure (e.g., formal webinars and workshops, virtual and in-person networking, and informal social media community); primary health care content (e.g., patient-centeredness, multimorbidity, home care, access, equity, and innovative research designs); and process (e.g., encouraging learning through explicit knowledge sharing and implicit mentorship opportunities). At the outset of this initiative, the working group determined that application of open-source virtual communication technology would be critical to build a meaningful network of colleagues, particularly because of the geographic distribution and the competing demands of completing graduate and postdoctoral training programs. As such, many of the capacity building activities (e.g., webinars and workshops) took place online, without any associated costs, and were recorded and available for trainees after the session.

**Table 1.** Characteristics of the trainee-led working group

Academic disciplines	Epidemiology, Family Medicine, Health Policy, Nursing, Social Work
Training levels	Master’s, doctoral, postdoctoral, early career researchers
Geographic locations	Alberta, Ontario, Québec, Manitoba, Prince Edward Island
Institutional affiliations	McMaster University, University of Alberta, Université de Sherbrooke, University of Manitoba, University of Ottawa, University of Toronto, Western University
CBPHC Team affiliations	ACCESS-MH (Atlantic Canada Children’s Effective Service & Strategies in Mental Health), ACHRU (Aging, Community and Health Research Unit), C-ChAMP (Canadian Chronic Disease Awareness and Management Program), FORGE-AHEAD (Transformation of Indigenous Primary Healthcare Delivery), iCOACH (Implementing Integrated Care for Older Adults with Complex Health), IMPACT (Innovative Models Promoting Access-to-Care Transformation), iPHIT (Innovation in Community-Based Primary Healthcare Supporting Transformation in the Health of First Nations and Rural/Remote Manitoba Communities), LHIV (Living with HIV), PACE in MM (Patient-Centred Innovations for Persons with Multimorbidity)

**Table 2.** Capacity building activities of the trainee-led working group

Online webinars and workshops	Nine sessions delivered by trainees and three sessions delivered by senior researchers
Health care innovation topics	Care delivery and culturally sensitive care delivery for aboriginal communities, home care case managers’ integrated care for older adults with multiple chronic conditions, wait times and patterns of care for cancer patients, enhancing home care eHealth application for stroke survivors with multiple chronic conditions, interprofessional education to support a team approach for older stroke survivors and family caregivers, implementing models of primary health care for older adults with complex needs
Research design topics	Measuring patient-centered care, pragmatic randomized controlled trials, adult patient/caregiver-oriented approaches to research, patient–provider communication
Knowledge translation	Fostering skills in collaborative abstract and manuscript writing, as well as collaborative poster and oral presentation development
Career advancement	Encouraging career building through increasing academic outputs (e.g., poster and oral presentations, invited online webinars, manuscript submission, and travel grant submissions) and networking opportunities
Social media	Promoting trainee research and career-oriented opportunities

## Responses from survey

A total of 27 trainees responded to the survey (response rate: 41.5%). Among these respondents, 44.0% were doctoral students, 22.0% were postdoctoral fellows, 19.0% were research coordinators, 19.0% were research assistants or research associates, 15.0% were Master’s students, and 4.0% were undergraduate students. Based on our definition of trainee, respondents may have identified as both graduate student and research coordinator, research assistant, or research associate. These trainees represented various clinical (family medicine, nursing, and social work) and nonclinical (epidemiology and health policy) disciplines. As seen in Table 3, participants identified key forms of learning in their roles within their CBPHC Team: (1) tacit knowledge; (2) knowledge gained from engaging in research activities; and (3) knowledge gained through teamwork within and across the CBPHC Teams. Tacit knowledge consisted of knowledge that was gained through implicit learning and personal observation, as opposed to knowledge that was gained in didactic workshops and webinars.

**Table 3.** Summary of responses from survey participants (*n* = 27)

Type	Activity	Have experienced, *n* (%)
Tacit	Establishing interdisciplinary research teams	23 (85.2)
	Leading and managing diverse research teams	20 (74.1)
	Moving research from proposal to implementation	19 (70.4)
Research	Importance of context in research design, result interpretation, and reporting	26 (96.3)
	Developing conference abstract development and submission	25 (92.6)
	Developing poster and oral conference presentations	25 (92.6)
Teamwork	Working with interdisciplinary research teams and the contributions from different disciplines	25 (92.6)
	Effective research team communication strategies	22 (81.5)
	Effective collaboration with external partners	20 (74.1)
Type	Activity	Would like to experience, *n* (%)
Tacit	Learning new teaching methods	11 (40.7)
	Learning about academic career development or promotion	8 (29.6)
	Leading and managing an interdisciplinary and geographically diverse research team	7 (25.9)
Research	Grant writing and submission	18 (66.7)
	Contributing to data collection	6 (22.2)
	Using appropriate methodology for different research questions	3 (11.1)
Teamwork	Effective approaches to conflict management	12 (44.4)
	Decision-making and consensus building in large research teams	7 (25.9)
	Rapport building and collaboration strategies	7 (25.9)

Among the three categories (tacit, research, and teamwork), research activities were experienced most by the respondents. More specifically, the majority of respondents indicated that they had learned the importance of context in research design, implementation, and reporting (96.3%); how to develop conference abstracts for submission (92.6%); and how to develop poster and oral conference presentations (92.6%). The trainees were then most likely to gain knowledge from teamwork activities including working with the contributions from different disciplines within a team (92.6%) and learning about effective team communication strategies (81.5%). Finally, the most common tacit learning was how to establish interdisciplinary research teams (85.2%) and how to lead and manage diverse teams (74.1%). In terms of the activities that the trainees would like to experience to build capacity, the top three activities were from each of the three categories (tacit, research, and teamwork). Respondents sought more experience in grant writing and submission (66.7%); effective approaches to conflict management (44.4%); and the opportunity to learn new teaching methods (40.7%). Trainees were also interested in learning more about academic career development or promotion (29.6%).

Based on the qualitative thematic analyses, both the competencies achieved during the CBPHC Signature Initiative and the areas for further capacity development are presented in Table 4. These competencies were aligned with the core competencies recommended by CIHR Canadian Health Services and Policy Research Alliance (Bornstein *et al.*, 2018). Competencies that were enhanced through the CBPHC capacity building initiatives included: analysis of data, evidence, and critical thinking; knowledge translation, communication, and brokerage; leadership, mentorship, and collaboration; interdisciplinary work; networking; research productivity; and peer-to-peer support and knowledge sharing. Overall, trainees indicated that they received advanced training in statistical or analysis software; learned skills for knowledge translation to different kinds of audiences; worked collaboratively with fellow trainees; developed and maintained productive relationships through virtual and in-person relationship building; presented and published completed and in-progress research; and participated in peer-to-peer support and knowledge sharing. In addition to advancing these competencies, trainees desired opportunities to expand skills in the leadership and management of an interdisciplinary research project. Trainees also expressed interest in developing experience and skills in group facilitation and to continue peer-to-peer support and knowledge sharing, importantly to address newly arising issues including career progression and establishing work–life balance.

**Table 4.** The core competencies recommended from the CIHR Canadian Health Services and Policy Research Alliance and acknowledged from the CBPHC trainee capacity building working group

Skills	Core competencies	CIHR description	Achieved through CBPHC	Further needs from trainees
Research and analytic skills	Analysis and evaluation of health and health-related policies and programs	The ability to effectively carry out formative and summative evaluation with strong links to organizational improvement and planning. Includes technical skills, contextual awareness, communication skills, analysis skills, and research skills.		
	Analysis of data, evidence, and critical thinking	The ability to collect, analyze, and use a wide range of data and to reflect critically on and incorporate theory and research evidence iteratively to clarify problems, frame options, and identify implementation considerations in both academic and nonacademic settings. Includes big data, administrative data, and economic data.	The ability to receive advanced training of statistical/analysis software for use in current and future research projects through in-person or online webinars and workshops.	The opportunities for advanced training on big data management and analyses, the use of logic models, and program evaluation approaches and the conduct of pragmatic trials in real-world and community settings.
	Understanding health systems and the policy-making process	Excellent knowledge of the Canadian and international health policy system from both academic and real-world perspectives.		
	Knowledge translation, communication, and brokerage	The ability to use multiple methods of communication and to communicate appropriately with different kinds of audiences.	The ability to learn the necessary skills for knowledge translation and integrated knowledge translation for a variety of audiences throughout a research project.	The opportunities to facilitate participant and patient engagement in research, as well as to conduct integrated knowledge translation with different kinds of audiences throughout a research project.
Professional skills	Leadership, mentorship, and collaboration	The ability to lead, organize, and support teams from various backgrounds to work together to achieve a specific outcome.	The ability to lead, organize, and support fellow trainees from various backgrounds and disciplines to work collaboratively together.	The opportunities to continue to build experience with leading organizing and supporting inter- and transdisciplinary teams to achieve a specific outcome.
	Project management	The ability to coordinate and organize all stages through to KTE of a project in an academic and nonacademic environment.		The opportunities to coordinate, organize, and lead a research project in academic and nonacademic environments, including aspects of recruitment, ethics approval, stakeholder engagement, and practical application of findings to policy-relevant issues.
	Interdisciplinary work	The ability to use effectively and to combine when appropriate methods and insights from multiple academic disciplines (e.g., humanities, social sciences, management, epidemiology, medicine, etc.).	The ability to effectively use and combine appropriate methods and insights from multiple academic disciplines (e.g., epidemiology, medicine, nursing, social sciences, etc.).	The opportunities to effectively use and combine appropriate methods and insights from academic and nonacademic disciplines.
	Networking	The ability to develop and maintain productive relationships within and outside of academia across the health system.	The ability to develop and maintain productive relationships in academia, as well as to learn from senior researchers and to network with potential mentors. This networking can be a combination of virtual and face-to-face relationship building.	The opportunities to continue to initiate and to maintain existing relationships in academia with peers, senior researchers, and other stakeholders.
	Dialogue and negotiation	The ability to work toward win–win outcomes and value-added results, including understanding other perspectives and how to respond.		The opportunities to build experience and skills in group facilitation and negotiation, including through engagement of participants/patients, community partners, coinvestigators, and decision-makers.
	Change management and implementation	The ability to plan, manage, and implement change, including: to communicate a clear vision for change; to lead people and organizations through change; to manage and implement successful transitions; and to evaluate and report on change.		
	Research productivity		The ability to present and share completed and in-progress research with peers through free online webinars, as well as to participate in manuscript preparation.	The opportunities to continue to present and publish research work, as well as to participate in and lead the generation of inter- and transdisciplinary funding applications.
	Peer-to-peer support and knowledge sharing		The ability to provide support and participate in networking with fellow trainees through a combination of virtual and face-to-face relationship building, as well as to share information about relevant capacity building and career building events, such as webinars and conferences.	The opportunities to continue peer-to-peer support and knowledge sharing to sustain relationships and collaborations, as well as to address new issues (e.g., career progression inside and outside of academia, how to establish work-life balance and personal well-being within academia, etc.).

CIHR = Canadian Institutes of Health Research; CBPHC = community-based primary health care; KTE = knowledge translation and exchange.

Throughout these capacity building initiatives, the working group experienced several process and data analysis challenges. The ongoing challenges included engaging all trainees from each of the 12-Teams (e.g., instead of a small group of already invested few); balancing information overload for busy trainees (e.g., emails, webinars, and workshops); and membership transition during the five-year funding program. Capacity building activities (particularly, webinars and workshops) needed to be accessible via internet or phone without any associated cost to the trainees and recorded and archived for those unable to participate in real time. Membership transition during this funding program impacted the total number of CBPHC trainees, the familiarity with the working group, and perhaps the response rate achieved for the final survey. The data analysis limitations were due to the small sample sizes as this did not allow for stratification of results by CBPHC Team, level of training, or academic discipline. Experiences from this capacity building initiative may have been different based on a trainee’s specific characteristics or affiliation with a specific CBPHC Team.

## Discussion

This paper describes the activities and outcomes of the capacity building activities for research trainees during the CBPHC Signature Initiative and facilitated by a capacity building committee and the trainee-led working group. The trainees within the working group had a diversity of characteristics (e.g., variety of disciplines, training levels, affiliations, and geographic locations) and included graduate students, postdoctoral fellows, early career researchers, and trainees who also worked as research staff. Activities included webinars and workshops delivered by both trainees and senior researchers to facilitate learning, as well as knowledge translation activities to share these capacity building efforts. In addition, each team provided experiences for the trainees to gain new research skills and experience. The completed surveys from trainees indicated the type of activities that either they had experienced or they would like to experience as part of the CBPHC Teams. The capacity building themes from these survey respondents indicated achievement in mandates of both the 12-Teams capacity building committee and the trainee-led working group aligned with the core competencies identified by the CIHR Canadian Health Services and Policy Research Alliance (Bornstein *et al.*, 2018).

Traditional models of mentorship consist of an experienced and established professional who provides support to a trainee in the form of guidance, encouragement, connections with appropriate networks, resources, and constructive feedback (Allen *et al.*, 2004; Berk *et al.*, 2005). In the case of the 12-Teams, this capacity building initiative fostered and facilitated a mentor/mentee network in a nationwide initiative that brought together researchers and trainees with expertise across a broad array of disciplines. Interestingly, this novel approach enabled trainees to mentor one another in a reciprocal manner, optimized individual trainees’ skill sets, built research capacity, created research networks, and enabled junior researchers to enact a mentorship role. This complementary role of supervisors and peers added a horizontal relationship among trainees, strengthening their connections (Detsky and Baerlocher, 2007). The survey findings also echo the mentorship literature on elements of successful relationships: reciprocity, respectful relationship with clear expectations, personal connection, and learner focused goals (Ploeg *et al.*, 2008; Straus *et al.*, 2014). As well, these findings reflect a recognized need for an enriched set of core competencies for trainees to ensure success for those with advanced education both inside and outside of academic careers (Bornstein *et al.*, 2018). We recommend that these enriched sets of core competencies are utilized in research funding proposals requiring a description of capacity development plans for trainees, particularly in the complex environment of CBPHC.

As described earlier, effective virtual communication was critical for building a meaningful network of colleagues in the context of geographic distance and competing demands. The working group had successes and challenges regarding activities for trainees. For example, the working group attempted to hold ‘virtual coffee or lunch breaks’ in which trainees would be able to join online for a chance to chat about their ongoing research work, career trajectories, or the shared experiences of being a trainee or early career researcher in primary health care. While there was some initial interest from trainees, this virtual strategy proved to be unsuccessful in terms of the number of attendees or ongoing interest in this opportunity. For example, this lack of success may have been due to the general nature of these get-togethers; the many competing demands of a trainee or early career researcher; or finding the right time for all interested trainees (although a poll was circulated to schedule the times with interested trainees and to account for time zone differences across the country). The bilingual nature of the CBPHC Signature Initiative meant that effective virtual communication was particularly important to ensure that bilingual trainees (whose first language was not English) had sufficient time to participate in discussions, as well as abstract and manuscript development. Indeed, this was a factor highlighted in facilitating the success of other mentorship initiatives (Byrne and Keefe, 2002). As well, both virtual and in-person relationship building can be important for trainees to foster strong collegial bonds as they move forward in their careers. These findings are consistent with previous successful approaches among other research groups in primary health care and suggest that events such as conferences could be integrated into mentor/mentee models as a means of enhancing relationships, allowing for varied networking opportunities and building collegiality among trainees (Coates *et al.*, 2004).

Although this CBPHC mentorship program has resulted in enhanced academic productivity (e.g., presentations and publications) and expanded collaborative networks of those trainees involved, these specific metrics have not been formally tracked during the process. Evaluating the number of peer-reviewed publications, government reports, and presentations at local, national, and international conferences led or co-authored by trainees, as a result of cross-team collaborations, could demonstrate the synergistic impacts of such approaches for other similar large multiteam training initiatives. In fact, a more formal evaluation could reinforce the value proposition for the career trajectories of academic trainees, such as through the expansion of networks of research collaborators, increased grant success, contributions to securing academic research positions, and practice or policy roles in CBPHC.

The trainee working group successfully engaged trainees from multiple CBPHC research teams across Canada, but there were some limitations. The working group leadership had trainees from seven of the CBPHC Teams, which provided a solid foundation for interdisciplinary peer mentorship and cross-institution networking. In addition, trainees from 7 of the 12-Teams participated at least one activity offered by the working group. However, despite providing diverse trainee activities and opportunities, there was no engagement from trainees on the remaining four teams (including the survey described herein). As mentioned earlier, 1 of the 12-Teams had opted to conduct their own trainee capacity building specific to their indigenous context and not participate in the larger trainee working group activities, so it has been excluded from this count. Since there were no survey responses from trainees on the other four teams, it is unknown what may have been the contributing factors to this lack of participation. For example, trainees working in larger research programs may have had ample opportunities for peer mentorship and feedback may have felt less compelled to engage with this initiative. Therefore, the working group was successful in achieving its trainee capacity building and mentorship mandate for trainees on the majority of the teams, but there is still opportunity to improve in future endeavors.

## Conclusions

This paper describes capacity building and mentorship activities developed and implemented for trainees during the CBPHC Signature Initiative and presents key insights from trainees on the mentorship that they received in a large, multiyear funding program in CBPHC. Importantly, this initiative fostered learning and collaboration across the 12-Teams. Building from the core competencies already identified in the existing literature, similar capacity building efforts should include the enriched set of competencies described herein to ensure that trainee needs and interests are represented during a research program.

The CBPHC Signature Initiative provided and facilitated trainees’ exposure to innovative research, expanded collaborative networks, and fostered synergies among junior researchers in primary health care. The combination of the advanced core competencies in trainee development, as well as the lessons learned from the trainee feedback, can inform dynamic and enhanced learning opportunities for early career researchers. To sustainably build primary health care research capacity, the research community must continue to develop and implement effective opportunities for capacity development and mentorship, as well as interdisciplinary, transdisciplinary, and cross-jurisdictional collaborations to support emerging researchers.

## References

[ref1] Allen TD , Eby LT , Poteet ML , Lentz E and Lima L (2004) Career benefits associated with mentoring for protégeé: a meta-analysis. Journal of Applied Psychology 89, 127–136. doi: 10.1037/0021-9010.89.1.127 14769125

[ref2] Ben Charif A , Hassani K , Wong ST , Zomahoun HTV , Fortin M , Freitas A , Katz A , Kendall CE , Liddy C , Nicholson K , Petrovic B , Ploeg J and Légaré F (2018) Assessment of scalability of evidence-based innovations in community-based primary health care: a cross-sectional study. Canadian Medical Association Open 6, e520–e527. doi: 10.9778/cmajo.20180143 PMC6221806 30389751

[ref3] Bennett S , Paina L , Kim C , Agyepong I , Chunharas S , McIntyre D and Nachuk S (2010) What must be done to enhance capacity for health systems research? Background paper for the Global Symposium on Health Systems Research. Montreux.

[ref4] Berk RA , Berg J , Mortimer R , Walton-Moss B and Yeo TP (2005) Measuring the effectiveness of faculty mentoring relationships. Academic Medicine 80, 66–71. doi: 10.1097/00001888-200501000-00017 15618097

[ref5] Bornstein S , Heritage M , Chudak A , Tamblyn R , McMahon M and Brown A (2018) Development of enriched core competencies for health services and policy research: training for stronger career readiness and greater impact. Health Services Research 53, 4004–4023. doi: 10.1111/1475-6773.12847 29527655 PMC6149358

[ref6] Braun V and Clarke V (2006) Using thematic analysis in psychology. Qualitative Research in Psychology 3, 77–101. doi: 10.1191/1478088706qp063oa

[ref7] Burgess JF , Menachemi N and Maciejewski ML (2018) Update on the health services research doctoral core competencies. Health Services Research 53 (Suppl 2), 3985–4003. doi: 10.1111/1475-6773.12851 29534339 PMC6149361

[ref8] Byrne MW and Keefe MR (2002) Building research competence in nursing through mentoring. Journal of Nursing Scholarship 34, 391–396. doi: 10.1111/j.1547-5069.2002.00391.x 12501744

[ref9] Canadian Institutes of Health Research (2013) CBPHC Objectives and Priority Areas. Retrieved 27 February 2018 from http://www.cihr-irsc.gc.ca/e/44765.html.

[ref10] Choi BC and Pak AW (2006) Multidisciplinarity, interdisciplinarity and transdisciplinarity in health research, services, education and policy: 1. Definitions, objectives and evidence of effectiveness. Clinical and Investigative Medicine 29, 351–364. 17330451

[ref11] Coates WC , Ankel F , Birnbaum A , Kosiak D , Broderick KB , Thomas S , Leschke R and Collings J (2004) SAEM Undergraduate Education Committee: the virtual advisor program: linking students to mentors via the world wide web. Academic Emergency Medicine 11, 253–255. 15001404 10.1111/j.1553-2712.2004.tb02205.x

[ref12] Detsky AS and Baerlocher MO (2007) Academic mentoring: how to give it and how to get it. Journal of the American Medical Association 297, 2134–2136. doi: 10.1001/jama.297.19.2134 17507350

[ref13] Dillman AD , Smyth JD and Christian LM (2014) Internet, phone, mail and mixed-mode surveys: the tailored design method, fourth edition New Jersey, USA: John Wiley & Sons.

[ref14] Green J and Thorogood N (2009) Qualitative methods for health research, second edition London, UK: SAGE Publications Ltd.

[ref18] Kendall C , Fitzgerald M , Kang RS , Wong ST , Katz A , Fortin M , Dionne E , Kuluski K , O’Brien MA , Ploeg J , Crowe L and Liddy C (2018) “Still learning and evolving in our approaches”: patient and stakeholder engagement among Canadian community-based primary health care researchers. Research Involvement and Engagement 4, 47–63. doi: 10.1186/s40900-018-0132-0 30524753 PMC6276251

[ref19] Lach H , Hertz J , Pomeroy S , Resnick B and Buckwalter K (2013) The challenges and benefits of distance mentoring. Journal of Professional Nursing 29, 39–48.

[ref20] Macinko J , de Fátima Marinho de Souza M , Guanais FC and da Silva Simões CC (2007) Going to scale with community-based primary care: an analysis of the family health program and infant mortality in Brazil, 1999–2004. Social Science & Medicine 65, 2070–2080. 17689847 10.1016/j.socscimed.2007.06.028

[ref21] Ploeg J , de Witt L , Hutchison B , Hayward L and Grayson K (2008) Evaluation of a research mentorship program in community care. Evaluation and Program Planning 31, 22–33. doi: 10.1016/j.evalprogplan.2007.10.002 18022693

[ref22] Sambunjak D , Straus SE and Marusic A (2006) Mentoring in academic medicine: a systematic review. Journal of the American Medical Association 296, 1103–1115. 16954490 10.1001/jama.296.9.1103

[ref26] Stewart M , Reid G , Brown JB , Burge F , Dicenso A , Watt S , McWilliam C , Beaulieu MD and Meredith L (2010) Development and implementation of training for interdisciplinary research in primary health care. Academic Medicine 85, 974–979. doi: 10.1097/ACM.0b013e3181dbe31f 20505396

[ref27] Stewart M , Wuite S , Ramsden V , Burge F , Beaulieu MD , Fortin M , Godwin M , Harris S , Reid G , Haggerty J , Brown JB , Thomas R and Wong S (2014) Transdisciplinary understandings and training on research: successfully building research capacity in primary health care. Canadian Family Physician 60, 581–582. 24925954 PMC4055331

[ref28] Straus SE , Johnson MO , Marquez C and Feldman MD (2014) Characteristics of successful and failed mentoring relationships: a qualitative study across two academic centers. Academic Medicine 88, 82–89. doi: 10.1097/ACM.0b013e31827647a0 PMC3665769 23165266

[ref29] Vaismoradi M , Turunen H and Bondas T (2013) Content analysis and thematic analysis: implications for conducting a qualitative descriptive study. Nursing & Health Sciences 15, 398–405. doi: 10.1111/nhs.12048 23480423

[ref31] Wong ST , Langton JM , Katz A , Fortin M , Godwin M , Green M , Grunfeld E , Hassani K , Kendall C , Liddy C , Ploeg J , Wodchis WP and Haggerty JL (2018) Promoting cross-jurisdictional primary health care research: developing a set of common indicators across 12 community-based primary health care teams in Canada. Primary Health Care Research & Development 6, 1–7. doi: 10.1017/S1463423618000518 PMC6476395 30396376

[ref32] World Health Organization (WHO) (1978) *Declaration of Alma Ata*. USSR: International Conference on Primary Health Care.

[ref33] Zea MC and Belgrave FZ (2009) Mentoring and research capacity-building experiences: acculturating to research from the perspective of the trainee. American Journal of Public Health 99, S163–169. 10.2105/AJPH.2008.149203 PMC2656593 19246665

